# The Potential of the Inclusion of *Prosopis farcta* Extract in the Diet on the Growth Performance, Immunity, Digestive Enzyme Activity, and Oxidative Status of the Common Carp, *Cyprinus carpio*, in Response to Ammonia Stress

**DOI:** 10.3390/ani15060895

**Published:** 2025-03-20

**Authors:** Morteza Yousefi, Hossein Adineh, Basim S. A. Al Sulivany, Ebrahim Gholamalipour Alamdari, Sevdan Yilmaz, Heba H. Mahboub, Seyyed Morteza Hoseini

**Affiliations:** 1 Department of Veterinary Medicine, RUDN University, Miklukho-Maklaya St., 117198 Moscow, Russia; myousefi81@gmail.com; 2 Department of Fisheries, Faculty of Agriculture and Natural Resources, Gonbad Kavous University, Gonbad Kavous 4971799151, Golestan, Iran; 3 Department of Biology, College of Science, University of Zakho, Zakho 42002, Duhok, Iraq; basim.ahmed@uoz.edu.krd; 4 Department of Plant Production, Faculty of Agriculture and Natural Resources, Gonbad Kavous University, Gonbad Kavous 4971799151, Golestan, Iran; eg.alamdari@gonbad.ac.ir; 5 Department of Aquaculture, Faculty of Marine Sciences and Technology, Canakkale Onsekiz Mart University, Canakkale 17100, Turkey; sevdanyilmaz@comu.edu.tr; 6 Department of Aquatic Animal Medicine, Faculty of Veterinary Medicine, Zagazig University, Zagazig P.O. Box 44511, Egypt; hhhmb@yahoo.com; 7 Inland Waters Aquatics Resources Research Center, Iranian Fisheries Sciences Research Institute, Agricultural Research, Education and Extension Organization, Gorgan 4916687631, Golestan, Iran

**Keywords:** feed additives, herbal supplements, nutrition, physiological responses, digestive enzymes

## Abstract

Herbal feed additives have benefits in aquaculture, as they can improve growth performance, welfare, and stress resistance. Hence, the effects of dietary *Prosopis farcta* extract (PFE) on the growth parameters, digestive enzyme activity, antioxidant defense, innate immunity responses, and resistance to ammonia stress in the common carp, *Cyprinus carpio*, were studied. Fish (15.14 ± 0.72 g) were fed on diets without the PFE (PFE0) or those fortified with 0.5% (PFE0.5), 1% (PFE1), or 2% (PFE2) PFE for 60 days and then subjected to ammonia stress for 24 h. The growth rate, feed efficiency, and amylase-, lipase-, and protease-specific activities in the PFE1 and PFE2 treatments showed significant elevations compared to these values in PFE0. The intestinal protease-specific activity significantly increased in all of the PFE treatments compared to that in the PFE0 treatment. Serum total protein and immunoglobulin significantly increased in the PFE1 treatment, whereas serum albumin and alternative complement activity significantly increased in the PFE2 treatment compared to these values in the PFE0 treatment. The PFE2 treatment significantly mitigated post-stress elevations in serum cortisol, glucose, malondialdehyde levels, alanine aminotransferase, and aspartate aminotransferase activities. The PFE1 treatment significantly mitigated post-stress elevations in CAT activity and decreases in SOD and lysozyme activity. In conclusion, 1–2% dietary PFE supplementation can improve the growth performance, health, and resilience to environmental stressors of the common carp.

## Simple Summary

Ammonia loading is a critical threat in the aquaculture industry, which suppresses the growth performance and welfare of fish. Dietary additives have been shown to be effective in reducing the negative effects of ammonia toxicity in aquaculture. In this study, dietary supplementation with 2% *Prosopis farcta* extract has been shown to promote digestive enzymes in and the growth performance of the common carp after 60 days of feeding. Moreover, this diet also mitigated physiological stress, immunosuppression, and oxidative stress in fish after exposure to ambient ammonia.

## 1. Introduction

The common carp, *Cyprinus carpio*, holds significant importance in aquaculture, ranking as the fourth most cultivated fish species after grass carp, silver carp, and tilapia. It accounts for 8.6% of the total fish production in inland waters [1]. As the global population continues to grow, the demand for food, particularly fish, has increased. However, the shortage of available areas suitable for fish production is a major challenge in aquaculture expansion. In response to this challenge, enterprises are seeking innovative methods to enhance the fish production per unit area [2]. Nonetheless, they face several obstacles, including a declining water quality, extended growth periods, disease outbreaks, and economic losses. A prevalent challenge in modern aquaculture is the accumulation of ammonia in the water, primarily caused by high stocking densities, low water flow rates, and suboptimal feed quality and practices [3]. Elevated ammonia levels can lead to increased energy expenditure, oxidative stress, and immunosuppression in fish, ultimately resulting in poor growth performance and overall health [3]. To ensure successful aquaculture practices, it is essential to address these issues.

One effective strategy for mitigating ammonia toxicity in fish is the application of functional feeds. These feeds are enriched with various additives known for their anti-stress, antioxidant, and immunostimulant properties, which can help counteract the adverse effects of ammonia exposure [3]. Herbal additives—such as whole plant materials, essential oils, and extracts—are particularly recognized for their antioxidant and immunostimulant benefits [4]. Research has shown that these herbal components can significantly alleviate the negative impacts of ammonia toxicity, especially concerning oxidative stress and immunosuppression in fish (as reviewed by Hoseini, Barbieri, Aydın, and Yousefi [3]). Given these promising findings, further investigation into the benefits of new herbal additives to mitigate ammonia toxicity in fish is warranted.

*Prosopis farcta* is a member of the Fabaceae family, recognized for its use as human food in the Mediterranean region [5] and as traditional medicine in the Middle East [6]. Studies have highlighted the extract’s diverse health benefits, including its anti-diabetic, anti-inflammatory, wound-healing [7], antifungal, anti-termite [8], antimicrobial, and antioxidant properties [9]. Notably, *P. farcta* extract (PFE) has shown inhibitory effects against fish pathogens in vitro [10]. Also, PFE is rich in gallic acid, vanillic acid, luteolin, and phloridzin [11], which contribute to boosting the immune function and disease resistance in fish [12,13,14,15].

The antioxidant, antimicrobial, and anti-inflammatory properties of PFE suggest that this extract may be a suitable candidate for aquaculture research. However, there is a lack of research on this topic; hence, this study aimed to fill this gap by investigating the growth performance, digestive enzyme activities, serum biochemistry, immune response, antioxidant status, and physiological changes in common carp fed diets supplemented with PFE at varying concentrations over a 60-day period.

## 2. Materials and Methods

### 2.1. Extraction of the Plant Material

*P. farcta* were collected from Azadshahr, Golestan, Iran. Initially, the dried fruits, leaves, and stems of *P. farcta* were milled, and 20 g of the milled materials was stirred with 200 mL of petroleum ether for 24 h at 37 °C. After filtering the extract through Whatman No. 1 filter paper, the filtrates were dried under reduced pressure using a rotary vacuum evaporator. The dried extracted materials were then re-extracted using methanol, freeze-dried, and analyzed (Supplementary Materials) using the HPLC-PDA-ESI-MS/MS method, as described previously [11]. The extract was kept at 4 °C until its further use in the fish feeds.

### 2.2. Preparation of the Diets and Administration of the Extract

A control diet without PFE supplementation (PFE0) and three diets supplemented with 0.5% (PFE0.5), 1% (PFE1), and 2% (PFE2) PFE were used in this study. These concentrations were chosen based on previous studies using herbal extracts in fish [16,17]. The dietary components listed in Table 1 were finely ground into powder and carefully sifted. The ingredients at appropriate amounts were then thoroughly mixed for 10 min, added to 0.3 L/kg water to make a dough, and pelleted using a meat grinder. Appropriate amounts of PFE were mixed with water before being added to the feed ingredient mixture. The pellets were dried overnight and stored at 4 °C until needed.

### 2.3. The Fish and Experimental Design

A total of 400 juvenile common carp were purchased from a local farm in Guilan province and transported to our laboratory at Gonbad Kavous University. The fish were placed into two separate 400 L concrete tanks (at a density of 1 fish/L) and fed on the PFE0 diet for a period of 14 days for acclimation. Then, 360 healthy juveniles (initial weight: 15.14 ± 0.72 g) were randomly selected and distributed into twelve 70 L tanks, ensuring three replicate tanks for each treatment group. Over a span of 60 days, the fish were fed their respective diets (mentioned above). Feeding was conducted manually three times daily (08.00, 13.00, and 18.00), with the total feed amounting to 2.5% of the fish’s biomass. The feed quantities were adjusted biweekly, and any uneaten feed and waste was removed daily. The tanks were continuously aerated, and the tank water was renewed daily at a rate of 50%.

The water’s physicochemical parameters were monitored throughout the experiment using digital apparatuses supplied by Hach Co. (a Hach HQ40d portable apparatus, Loveland, CO, USA) and Wagtech Co. (a Wagtech photometer 7100, Berkshire, UK). The water’s temperature, dissolved oxygen, pH, unionized ammonia, total alkalinity, and electro-conductivity were 24.36 ± 0.60 °C, 6.54 ± 0.42 mg/L, 7.70 ± 0.12, 0.017 ± 0.004 mg/L, 378.83 ± 8.12 mg CaCO_3_/L, and 895.97 ± 5.59 μs/cm, respectively (mean ± standard deviation).

### 2.4. Fish Biometry and Sampling

The biomass and total feed intake of each tank were determined at the termination of the experiment to calculate the growth performance parameters and survival as follows:

Weight gain (WG, %) = (FW − IW) × 100/IW

Specific growth rate (SGR, %/day) = 100 × [(ln FW − ln IW)/d]

Feed conversion ratio (FCR) = FI/(FW − IW)

Survival rate (SR, %) = 100 × (Final specimens per tank/Initial specimen per tank)

where FW, final weight; IW, initial weight; d, number of rearing days; and FI, feed intake.

The fish underwent 24 h fasting for sampling. Three fish were randomly selected from each tank and anesthetized using 200 mg/L of clove extract [18]. Blood samples were collected from the caudal vein, allowed to clot at room temperature, and subsequently centrifuged at 5000× *g* for 10 min at 4 °C to separate the serum. Additionally, the entire intestines of the fish were removed for further analysis of the digestive enzyme activity. All samples were promptly frozen in liquid nitrogen and stored at −80 °C for future studies.

### 2.5. Ambient Ammonia Exposure

Following the initial sampling procedure, the water flow in the tanks was ceased, and the fish were exposed to 0.5 mg/L of unionized ammonia nitrogen for 24 h according to the method described previously for common carp [19]. The unionized ammonia concentration was calculated based on the water temperature and pH [20]. Accordingly, 106 mg/L of ammonium chloride was added to the tank water. After 24 h of exposure, a second set of blood samples was taken from all tanks (from 3 fish per tank).

### 2.6. Analysis

#### 2.6.1. Digestive and Antioxidant Enzymes

To prepare the enzyme extracts, the intestine samples were homogenized using Tris-HCl buffer (50 mM; pH 7.5) and a homogenizer (Heidolph^®^ Silentcrusher-m, Schwabach, Germany) for 1.5 min. Then, the supernatant was separated from impurities using a centrifuge at 10,000× *g* for 20 min. The upper solution was kept at −80 until the digestive-enzyme-specific activity was measured. P-nitrophenyl myristate was used as the substrate for the lipase-specific activity assay at a pH of 9.0 and a temperature of 30 °C, and the release of one pM of p-nitrophenol per min was considered to be one unit of lipase [21]. The amylase-specific activity was determined using soluble starch as the substrate at a pH of 6.9 and at 20 °C. Each unit of amylase activity was defined as the amount of enzyme required to release one mg of maltose within 3 min [22]. Protease activity was measured using azocasein as the substrate at a pH of 9.5 and at 25 °C. The optical densities of the samples were converted into units of protease activity using a standard curve from pure protease [23]. The soluble protein of the enzyme extracts was measured according to Lowry et al. [24].

Commercial kits from Zellbio Co. (Deutschland, Germany) were used to measure the serum superoxide dismutase (SOD)-, catalase (CAT)-, glutathione peroxidase (GPX)-specific activities and malondialdehyde (MDA) concentration, as suggested for this fish species [25]. SOD activity was determined using a pyrogallol autoxidation method, and one unit of the enzyme was equivalent to the amount required to inhibit autoxidation by 50%. CAT activity was determined using hydrogen peroxide as the substrate, and one unit of enzyme activity was taken as an amount that decomposed one molecule of hydrogen peroxide. GPX activity was determined using Elman’s reagent, and one unit of the enzyme was equal to an amount that oxidized 2 molecules of reduced glutathione. MDA was determined based on the thiobarbituric acid reaction at 95 °C.

#### 2.6.2. Biochemical Markers

The activity of serum aspartate aminotransferase (AST) and alanine transferase (ALT) was assessed using colorimetric test kits from Zist Chem Co. (Tehran, Iran), according to the manufacturer’s protocols. The ALT assay was performed using L-Alanine and 2-Oxoglutarate as the substrates, and the AST assay was performed using aspartate and α-ketoglutarate as the substrates. One unit of ALT and AST activity was defined as a 0.00057 decrease in the optical density per minute. The serum glucose concentrations were quantified using the glucose oxidase method, using a commercial kit supplied by Zist-Chem Co. (Tehran, Iran). Serum cortisol was evaluated using an ELISA kit from IBL GmBH (Magdeburg, Germany), with the measurements taken at 450 nm, as previously reported by Yousefi et al. [26] for the same fish species.

#### 2.6.3. Innate Immune Responses

The total protein (TP) and albumin (ALB) levels in the serum were determined using commercial kits provided by Zist Chem Co. (Tehran, Iran) at 546 nm. Lysozyme activity was assessed using a turbidimetric test, as described by Ellis [27], where 1 mL of a *Micrococcus luteus* suspension was mixed with 25 μL of serum, and the mean reduction in the optical density per minute was recorded over five min at 450 nm. Each 0.001 decrease in the optical density was considered to be one unit of lysozyme activity. To evaluate the alternative pathway activity of the complement system (ACH50), sheep erythrocytes in veronal buffer (containing gelatin, EGTA, and Mg^2+^) were used to measure the hemolytic activity of the samples [28]. Finally, the serum total immunoglobulin (Ig) levels were determined according to Siwicki and Anderson [29], where the Ig content was calculated based on the difference in the protein quantity before and after adding polyethylene glycol to the serum samples.

### 2.7. Statistical Analysis

To conduct a statistical analysis of the data, SPSS software (version 20) was utilized. Prior to the analysis, the data’s normality and the homogeneity of variance were assessed using the Shapiro–Wilk and Levene’s tests, respectively. When these conditions were satisfied, the impact of the PFE on the growth and digestive enzyme data were analyzed using a one-way ANOVA, followed by Duncan’s multiple range tests. Additionally, a two-way ANOVA and Duncan’s multiple range tests were performed to evaluate the effects of the PFE and ammonia stress on immune, biochemical, and antioxidant serum markers. A significance level of *p* < 0.05 was established, and the results are presented as the mean ± SD.

## 3. Results

There was no mortality among the treatments during the feeding trial. Dietary PFE supplementation significantly improved the WG, SGR, and FI, and the highest values were observed in the PFE1 and PFE2 treatments. A significant elevation in FW and decrease in the FCR were observed in the PFE1 and PFE2 treatments (Table 2).

**Figure 1:**
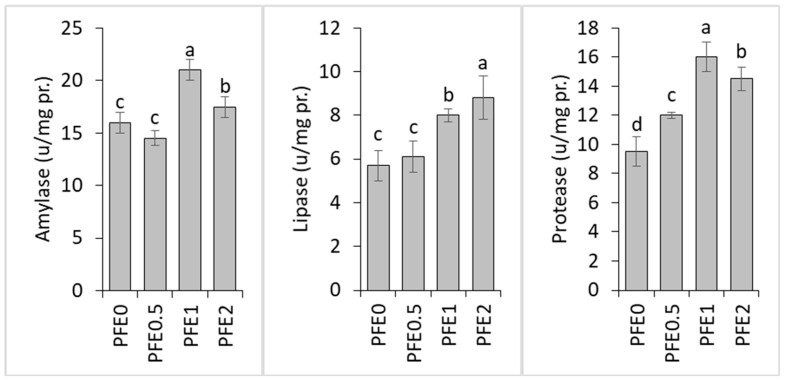
Digestive enzyme-specific activities of common carp fed diets supplemented with 0.5–2% PFE for 60 days. Dissimilar letters above the bars represent significant differences among the experimental groups (Duncan’s test; *n* = 3, *p* < 0.001).

The PFE1 and PFE2 treatments had significantly higher intestinal amylase- and lipase-specific activities compared to these values in PFE0. The PFE1 treatment had the highest amylase-specific activity, while the PFE2 treatment had the highest lipase activity. The intestinal protease levels in the PFE-treated fish were significantly higher than those in the PFE0 treatment, and the highest specific activity was observed in the PFE1 treatment (Figure 1).

According to Table 3, a significant interaction between dietary PFE and ammonia exposure was observed for serum cortisol (*p* < 0.001), glucose (*p* < 0.001), AST (*p* < 0.001), and ALT (*p* < 0.001), indicating that the effect of PFE on these parameters depended on the presence of ammonia stress. Before ammonia exposure, dietary PFE resulted in significant decreases in serum cortisol, glucose, AST, and ALT (PFE1- PFE2) compared to these values in PFE0. The exposure of fish to ammonia stress resulted in elevations in serum cortisol, glucose, AST, and ALT; however, the PFE treatments showed lower elevations compared to those in the PFE0 treatment. The lowest glucose, AST, and ALT values were observed in the PFE1 and PFE2 treatments before ammonia exposure. The lowest cortisol and AST values were observed in the PFE1 and PFE2 treatments after ammonia exposure. At this time, the lowest glucose and ALT levels were observed in the PFE2 treatment.

Table 4 shows the serum antioxidant parameters of the fish before and after ammonia exposure. There were interactions between dietary PFE and ammonia exposure in terms of their effects on serum SOD, GPX, and MDA (*p* < 0.001). The serum SOD in the PFE1 and PFE2 treatments was significantly higher than that in PFE0 before and after ammonia exposure. These treatments showed significantly lower serum MDA levels compared to those in the PFE0 treatment before and after ammonia exposure. All of the PFE treatments had significantly higher serum GPX levels compared to those in the PFE0 treatment, before and after ammonia exposure. Serum SOD, GPX, and MDA showed significant elevations after ammonia exposure. Dietary PFE and exposure to ammonia had significant effects on serum CAT activity; an increase in dietary PFE significantly decreased the serum CAT activity, whereas ammonia exposure significantly increased it (*p* < 0.001).

The serum immunological parameters are shown in Table 5. There was an interaction effect of dietary PFE and exposure to ammonia on serum lysozyme (*p* < 0.001). The serum lysozyme activities in the PFE treatments were significantly higher than those in the PFE0 treatment, and the highest lysozyme values were observed in the PFE1 treatment, before and after ammonia exposure. Dietary PFE and exposure to ammonia had significant effects on serum total Ig, ACH50, and total protein and albumin levels. These parameters showed significant increases due to dietary PFE supplementation but showed significant decreases after the ammonia challenge.

## 4. Discussion

This study demonstrated that the addition of PFE to common carp feed enhanced the fish’s growth, feed efficiency, and intestinal digestive enzymes. Hence, improved nutrient digestion and absorption in fish fed PFE-supplemented diets may be one of the reasons for the better growth performance (as reviewed by Citarasu [30] and Pau et al. [31]). Moreover, stress leads to cortisol elevation and an increase in energy expenditure, which suppress fish growth [32]. Previous research has indicated that some plant extracts have anti-stress effects in fish raised in aquaculture facilities, enabling them to utilize the energy from their feed better and thereby improving their growth performance [33,34,35]. As the PFE decreased their serum cortisol and glucose, it is speculated that the improved growth performance in the fish in the PFE treatments may have been partially related to lower basal energy expenditure.

Serum cortisol and glucose levels serve as crucial indicators for monitoring stress in fish, which increase under unfavorable physicochemical conditions in the fish-rearing water [36]. Elevated levels of serum cortisol and glucose are typically observed in fish exposed to high water ammonia concentrations, enabling them to cope with an increment in energy demand [37]. In the carp fed the diets supplemented with PFE, a notable decrease in their serum cortisol and glucose levels was observed both before and after exposure to ammonia stress. These results suggest an improvement in the fish’s welfare and the mitigation of ammonia-induced stress. It was previously found that the elevations in serum glucose and cortisol after ammonia exposure decreased in common carp when they were fed diets containing other herbal supplements, such as garlic meal [38] and *Tanacetum balsamita* essential oil [26], which partially supports the present findings.

Elevated serum levels of AST and ALT in fish are commonly recognized as indicators of liver dysfunction or stressful conditions. When these enzymes increase in the liver (due to stress and the activation of gluconeogenesis) or the hepatocytes are damaged, they leak into the circulation [39]. In this study, the fish’s serum AST and ALT increased after ammonia exposure, but the PFE decreased such elevations. Similarly, previous research has shown that incorporating beneficial herbal supplements into carp feed can lead to reductions in their serum AST and ALT levels prior to and following ammonia exposure [19,38,40]. Numerous studies have documented the hepatoprotective and anti-stress effects of medicinal plants when added to fish feeds (as reviewed by Ciji and Akhtar [32]). Although such data are not available for *P. farcta* in fish, various types of *P. farcta* extract have shown hepatoprotective characteristics in non-fish species [41,42].

Elevations in serum lysozyme and ACH50 activities and total Ig, total protein, and albumin levels are typically indicators of robust immune strength in fish [43]. In this study, the carp fed the PFE-supplemented feeds exhibited increases in all of the immune-related parameters tested. Such improvements were also evident after ammonia exposure, when the fish experienced immunosuppression. These results affirm the immunomodulatory effects of PFE in common carp and align with previous studies indicating that various herbal supplements can enhance the immunological parameters in common carp following ammonia stress [26,44]. PFE contains bioactive compounds which may be responsible for its immunomodulation effects [11]. Supporting this, previous studies have shown that gallic acid [12], vanillic acid [13], luteolin [14], and phloridzin [15] contribute to the enhancement of immune function and disease resistance in fish fed various phytobiotic-supplemented diets.

Ammonia exposure leads to oxidative stress, partially due to an increase in cell respiration (stressful conditions) and the activation of oxidative enzymes, such as xanthine oxidase and aldehyde oxidase [45]. This was evident in the present study, as the SOD, CAT, and GPX activities increased, along with an increase in MDA levels, in the fish after ammonia exposure. Enzymatic antioxidant mechanisms, including SOD, CAT, and GPX, primarily function to detoxify and mitigate the harmful effects of reactive oxygen species and free radicals [46]. Elevated levels of MDA serve as a marker for lipid peroxidation and oxidative stress [47]. SOD is responsible for the neutralization of superoxide ions, which results in the formation of a less reactive compound, hydrogen peroxide [48]. When it is found at low concentrations, hydrogen peroxide is neutralized into water by GPX, whereas high concentrations of hydrogen peroxide are neutralized by CAT [49]. Accordingly, the present results showed that PFE improved the antioxidant capacity in the carp, as SOD and GPX increased and CAT decreased, suggesting a low amount of hydrogen peroxide accumulation. Under these conditions, oxidative stress decreases, characterized by lower MDA levels. These findings suggest that PFE has antioxidant properties. Similarly, previous research has indicated that fish receiving herbal supplements demonstrate enhanced antioxidant defense mechanisms in response to ammonia exposure [19,38,40,44]. Although there is currently no literature specifically addressing the antioxidant effects of PFE in fish, studies have shown that the oxidative stress is reduced and the antioxidant capacity is improved in diabetic rats treated with PFE [50].

## 5. Conclusions

In conclusion, dietary PFE can increase the activity of digestive enzymes, which may be the reason for the growth promotion in the common carp. This study showed that PFE improved the antioxidant and immunological parameters and decreased the stress following ammonia exposure in these fish. Based on the results, 1–2% dietary PFE supplementation is recommended as a feed additive to promote carp growth and provide protection during periods of heightened ammonia toxicity risk.

## Figures and Tables

**Table 1:** Feed ingredients and chemical compositions of different diets.

Ingredients (g/kg Diet)	PFE0	PFE0.5	PFE1	PFE2
Kilka fishmeal (72.72% CP)	100	100	100	100
Poultry slaughterhouse by-product (58.5% CP)	200	200	200	200
Soybean meal (46.78% CP)	230	230	230	230
Wheat meal (14.52% CP)	389	384	379	369
Corn flour (7.77% CP)	50.0	50.0	50.0	50.0
Fish oil	7.00	7.00	7.00	7.00
Soybean oil	7.00	7.00	7.00	7.00
Lysine	7.00	7.00	7.00	7.00
Methionine	5.00	5.00	5.00	5.00
Vitamin premix 1	2.50	2.50	2.50	2.50
Mineral premix 1	2.50	2.50	2.50	2.50
PFE	-	5.00	10.0	20.0
Total	1000	1000	1000	1000
Dry matter	89.22	89.17	89.13	89.04
Crude protein (%)	35.30	35.30	35.29	35.27
Crude fat (%)	5.78	5.78	5.78	5.78
Crude ash (%)	5.88	5.88	5.88	5.87
Energy (kcal/kg)	4049.19	4047.23	4045.27	4041.35

1. See Supplementary Materials “Table S2”.

**Table 2:** Growth performance, feed efficiency, and survival rate of common carp fed diets supplemented with 0.5–2% PFE for 60 days. Different letters in a row show a significant difference among the treatments (Duncan’s test; *n* = 3).

	PFE0 (Control)	PFE0.5	PFE1	PFE2	*p*-Value
IW (g)	14.9 ± 0.41	14.9 ± 0.20	15.4 ± 0.44	15.2 ± 0.50	0.379
FW (g)	36.7 ± 1.79 b	38.3 ± 0.75 b	44.4 ± 1.15 a	44.7 ± 2.23 a	<0.001
WG (%)	146 ± 3.59 c	157 ± 1.75 b	188 ± 1.77 a	194 ± 5.15 a	<0.001
SGR (%/d)	1.50 ± 0.02 c	1.58 ± 0.01 b	1.76 ± 0.01 a	1.80 ± 0.03 a	<0.001
FI (g/fish)	39.4 ± 0.24 a	41.8 ± 0.34 b	43.6 ± 0.15 c	43.8 ± 0.22 c	<0.001
FCR	1.81 ± 0.10 a	1.79 ± 0.03 a	1.51 ± 0.05 b	1.49 ± 0.09 b	0.001
Survival (%)	100	100	100	100	

IW: initial weight; FW: final weight; SGR: specific growth rate; WG: weight gain; FI: feed intake; FCR: feed conversation ratio.

**Table 3:** Biochemical parameters in common carp fed diets supplemented with 0–2% PFE for 60 days and exposed to 0.5 mg/L of water ammonia for 24 h. Lowercase letters within a column show significant differences among different levels of dietary PFE (Duncan’s test), whereas the asterisks show a significant difference compared to the basal values (*n* = 3).

	Cortisol (ng/mL)	Glucose (mg/dL)	AST (U/L)	ALT (U/L)
Basal				
PFE0	57.87 ± 4.99 a	80.06 ± 2.43 a	278.47 ± 3.75 a	14.28 ± 0.68 a
PFE0.5	49.34 ± 2.78 b	75.40 ± 1.50 b	254.58 ± 3.34 b	13.36 ± 0.66 a
PFE1	47.42 ± 2.95 b	71.21 ± 1.98 c	241.69 ± 5.97 c	10.23 ± 0.38 b
PFE2	47.71 ± 3.26 b	69.62 ± 2.53 c	238.80 ± 2.11 c	11.24 ± 0.60 b
Stressed				
PFE0	135.97 ± 3.44 a*	165.02 ± 2.83 a*	399.95 ± 5.33 a*	23.76 ± 0.73 a*
PFE0.5	91.25 ± 2.88 b*	112.59 ± 1.73 b*	300.01 ± 4.32 c*	18.60 ± 0.69 b*
PFE1	80.21 ± 5.18 c*	98.99 ± 3.63 c*	327.19 ± 4.22 b*	12.12 ± 0.54 d*
PFE2	76.07 ± 3.45 c*	90.50 ± 2.95 d*	322.37 ± 4.41 b*	14.28 ± 0.71 c*
Two-way ANOVA				
PFE	*p* < 0.001	*p* < 0.001	*p* < 0.001	*p* < 0.001
Stress	*p* < 0.001	*p* < 0.001	*p* < 0.001	*p* < 0.001
Interaction	*p* < 0.001	*p* < 0.001	*p* < 0.001	*p* < 0.001

AST: aspartate aminotransferase; ALT: alanine aminotransferase.

**Table 4:** Serum antioxidant enzyme activity and MDA content in common carp fed diets supplemented with 0–2% PFE for 60 days and exposed to 0.5 mg/L of water ammonia for 24 h. Lowercase letters within a column show significant differences among different levels of dietary PFE (Duncan’s test), whereas asterisks show a significant difference compared to the basal values (*n* = 3).

	SOD (U/mL)	GPX (U/mL)	Catalase (U/mL)	MDA (µmol/L)
Basal				
PFE0	48.52 ± 1.36 c	194.18 ± 8.69 b	75.99 ± 2.11	67.65 ± 2.31 a
PFE0.5	49.55 ± 0.53 c	217.47 ± 5.84 a	68.23 ± 1.55	63.53 ± 1.65 a
PFE1	57.30 ± 1.05 a	222.60 ± 7.98 a	64.72 ± 1.62	57.71 ± 1.38 b
PFE2	55.24 ± 0.93 b	223.78 ± 6.58 a	61.12 ± 1.20	53.11 ± 3.98 b
Stressed				
PFE0	45.58 ± 1.00 c*	240.07 ± 4.27 b*	81.66 ± 1.63	87.91 ± 2.30 a*
PFE0.5	55.57 ± 0.64 c*	255.27 ± 6.13 a*	73.36 ± 0.94	76.44 ± 1.68 a*
PFE1	60.11 ± 1.04 a*	258.49 ± 5.12 a*	66.21 ± 3.83	59.32 ± 3.31 b
PFE2	59.80 ± 0.90 b*	299.71 ± 3.94 a*	65.40 ± 2.02	62.32 ± 1.71 b*
Two-way ANOVA				
PFE	*p* < 0.001	*p* < 0.001	*p* < 0.001	*p* < 0.001
Stress	*p* < 0.001	*p* < 0.001	*p* < 0.001	*p* < 0.001
Interaction	*p* < 0.001	*p* < 0.001	*p* = 0.327	*p* < 0.001
Stress effects				
Basal	NA	NA	67.52 ± 5.92	NA
Stressed	NA	NA	71.66 ± 7.14	NA
Diet effects				
PFE0	NA	NA	78.83 ± 3.54	NA
PFE0.5	NA	NA	70.80 ± 3.03	NA
PFE1	NA	NA	65.47 ± 2.76	NA
PFE2	NA	NA	63.26 ± 2.78	NA

SOD: superoxide dismutase; GPX: glutathione peroxidase; CAT: catalase; MDA: malondialdehyde. NA: not applicable.

**Table 5:** Innate immunity responses in common carp fed diets supplemented with 0–2% PFE for 60 days and exposed to 0.5 mg/L of water ammonia for 24 h. Lowercase letters within a column show significant differences among different levels of dietary PFE (Duncan’s test), whereas asterisks show a significant difference compared to the basal values (*n* = 3).

	Lysozyme (U/mL)	Total Ig (mg/mL)	ACH50 (U/mL)	Total Protein (g/dL)	Albumin (g/dL)
Basal					
PFE0	34.96 ± 0.72 c	15.70 ± 0.42	130.00 ± 4.08	2.58 ± 0.13	1.02 ± 0.03
PFE0.5	37.31 ± 0.69 b	19.58 ± 0.45	136.03 ± 3.90	3.24 ± 0.07	1.33 ± 0.06
PFE1	39.05 ± 0.89 a	20.66 ± 1.01	137.24 ± 2.12	3.71 ± 0.16	1.39 ± 0.04
PFE2	38.14 ± 0.56 ab	19.78 ± 0.87	141.62 ± 4.05	3.46 ± 0.12	1.38 ± 0.05
Stressed					
PFE0	28.73 ± 0.83 c*	11.94 ± 0.83	125.51 ± 3.74	2.27 ± 0.14	0.99 ± 0.11
PFE0.5	33.00 ± 1.04 b*	16.48 ± 0.54	131.75 ± 3.27	2.94 ± 0.08	1.07 ± 0.06
PFE1	35.16 ± 0.96 a*	18.67 ± 0.37	135.02 ± 4.65	3.15 ± 0.03	1.17 ± 0.05
PFE2	35.94 ± 0.92 ab*	18.28 ± 0.80	137.88 ± 3.24	2.91 ± 0.09	1.24 ± 0.12
Two-way ANOVA					
PFE	*p* < 0.001	*p* < 0.001	*p* < 0.001	*p* < 0.001	*p* < 0.001
Stress	*p* < 0.001	*p* < 0.001	*p* = 0.027	*p* < 0.001	*p* < 0.001
Interaction	*p* = 0.007	*p* = 0.051	*p* = 0.950	*p* = 0.101	*p* = 0.082
Stress effects					
Basal	NA	18.93 ± 2.09	136.22 ± 5.33	3.24 ± 0.45	1.28 ± 0.16
Stressed	NA	16.34 ± 2.84 *	132.54 ± 5.78 *	2.82 ± 0.35 *	1.12 ± 0.13 *
Diet effects					
PFE0	NA	13.82 ± 2.14 a	127.75 ± 4.28 a	2.43 ± 0.21 a	1.01 ± 0.08 a
PFE0.5	NA	18.03 ± 1.76 b	133.89 ± 3.99 b	3.09 ± 0.18 b	1.20 ± 0.15 b
PFE1	NA	19.66 ± 1.29 c	136.13 ± 3.46 bc	3.43 ± 0.32 c	1.28 ± 0.13 bc
PFE2	NA	19.03 ± 1.11 c	139.75 ± 3.87 c	3.19 ± 0.32 b	1.31 ± 0.12 c

Ig: immunoglobulin; ACH50: alternative complement activity; NA: not applicable.

## Data Availability

The original contributions presented in this study are included in the article/Supplementary Materials. Further inquiries can be directed to the corresponding author(s).

## Supplementary Materials

The following supporting information can be downloaded at https://www.mdpi.com/article/10.3390/ani15060895/s1. Table S1: Phenolics identified in different parts of *P. farcta* using HPLC-PDA-ESI-MS/MS; Table S2: Compositions of vitamin and mineral premixes; Table S3: Raw data.
