# Supplementary material for: The Potential of the Inclusion of Prosopis farcta Extract in the Diet on the Growth Performance, Immunity, Digestive Enzyme Activity, and Oxidative Status of the Common Carp, Cyprinus carpio, in Response to Ammonia Stress
Source: Animals (Basel). 2025 Mar 20;15(6):895. doi: 10.3390/ani15060895 (PMC11939293; doi:10.3390/ani15060895)
Supplement: Supplementary file 1 [file animals-15-00895-s001.zip › Table S1.pdf]

Table S1: Phenolic identified in different parts of *P. farcta* using HPLC-PDA-ESI-MS/MS.

N0.	Compound	Molecular Weight	$[M-H]^-$ (m/z)	MS^2 Fragments	R_t (min)/ Intensity (E^n)		
					Fruits	Leaves	Stems
1	Acacetin	284	283	268	25.4/ 1.7E5	-	-
2	Apigenin	270	269	269, 241, 225, 201	24.5/ 3.5 E5	23.8/ 1.8E5	24.1/ 1.7E5
3	Luteolin	286	285	269	22.6/ 2.1E5	22.2/ 3.9E5	22.6/ 3.8E5
4	Chrysoeriol-methyl ether	314	313	298, 283	23.3/ 1.9E5	24.6/ 2.6E5	23.6/ 8.3E4
5	7,4'-Dihydroxy-3'-methoxyflavone	284	283	268, 255	26.7/ 1.2E5	26.1/ 1.4 E5	27.1/ 1.6E5
6	Quercetin	302	301	151, 158	21.5/ 1.3 E5	20.9/ 1.3E5	21.7/ 1.1E5
7	Kaempferide	300	299	284	23.7/ 1.2E5	23.1/ 2.9E5	23.6/ 8.6E4
8	Tamarixetin	316	315	300	22.2/ 1.7 E5	21.7/ 1.6E5	22.4/ 2.0E5
9	Quercetin-3-methyl ether	316	315	300	-	21.2/ 1.5E5	21.7/ 2.8E5
10	Isorhamnetin	316	315	300	-	20.7/ 1.2E5	19.5/ 7.6E4
11	Formononetin	268	267	239, 211	27.5/ 1.4 E5	26.0/ 2.5E5	27.9/ 2.5E5
12	Apigenin -7-O- rutinoside	578	577	415, 269	11.8/ 1.2E5	11.5/ 1.2E5	
13	Apigenin-7-O-glucoside	432	431	269	19.5/ 1.2E5	18.9/ 1.2E5	-

14	Dihydrokaempferol hexoside	450	449	329, 287, 269, 259, 243, 199	17.3/ 9.0E4	16.9/ 1.3E5	-
15	Dihydrokaempferol-3-O-rhamnoside	450	449	329, 287, 269, 259, 243, 199	17.5/ 5.5E4	-	-
16	Diosmetin-7-O-rutinoside	608	607	299, 284	10.7/ 7.2E4	10.9/ 1.4E5	11.5/ 8.4E4
17	Tricin-7-O-glucoside	492	491	476, 329, 314	-	15.2/ 1.0E5	14.2/ 1.2E5
18	Linarin	592	591	445, 283, 268	12.1/ 8.4E4	12.1/1.0E5	12.6/ 8.8E4
19	Acacetin-7-O-glucoside	446	445	283, 268	21.3/ 8.5E4	20.8/ 5.4E4	-
20	Chrysoeriol 7-O-glucoside	462	461	446, 341, 299, 284	13.8/ 8.4E4	13.5/ 1.7E5	14.2/ 8.7E4
21	Cynaroside	448	447	285	13.1/ 1.1E5	12.8/ 9.5E4	14.4/ 6.9E4
22	Astragalin	448	447	285	14.6/ 1.4E5	14.4/ 1.5E5	14.9/ 8.6E4
23	3', 4'-dimethoxyluteolin-7-O-rutinoside	622	621	313, 298, 283	11.1/ 1.2E5	10.6/ 1.6E5	11.4/ 9.7E4
24	Nicotiflorin	594	593	285	11.5/ 7.4E4	11.0/ 1.0E5	11.7/ 3.7E5
25	Quercetin 3,3'-dimethyl ether 7-rutinoside	638	637	329, 314, 299	9.1/ 1.5E5	8.8/ 1.5E5	9.4/ 7.7E4
26	Hyperoside	464	463	310	12.9/ 2.0E5	12.3/ 2.4E5	-
27	Isoquercitrin	464	463	310	13.3/ 1.3E5	12.7/ 1.2E5	-

28	Rutin	610	609	301, 271	8.7/ 7.2E4	8.1/ 1.8E5	8.9/ 1.3E5
29	Narcissin	624	623	477, 315	10.2/ 2.0E5	9.8/ 1.7E5	10.9/ 1.5E5
30	Isorahmnetin 3-O-glucoside	478	477	315	-	-	14.1/ 7.6E4
31	Vicenin-2	594	593	575, 503, 473, 383, 353	12.5/ 9.8E4	12.0/ 1.5E5	12.3/ 4.0E5
32	Iso-orientin	448	447	357, 327	14.2/ 1.6E5	13.8/ 2.6E5	14.6/ 7.2E4
33	Vitexin	432	431	413, 341, 311, 269	17.9/ 1.2E5	-	18.1/ 7E4
34	Isovitexin	432	431	341, 311, 413, 353	18.1/ 8.1E4	-	18.5/ 5.2E4
35	Vitexin-2"-O-rhamnoside	578	577	457, 431, 413, 311, 293	-	11.7/ 7.6E4	-
36	Syringic acid-O-hexoside	360	359	197	0.2/ 1.2E5	1.0/ 2.7E5	0.8/ 9.7E4
37	Protocatechuic -acid-4-glucoside	316	315	153, 109	-	-	2.2/ 2 E5
38	Dihydrocaffeic acid 3-sulfate	262	261	181	5.5/ 2.1 E5	4.9/ 3.4E5	5.8/ 1.2E5
39	Methylgallic acid-O-sulphate	264	263	284, 183, 167	6.6/ 2.1 E5	6.4/ 1.1E5	7.1/ 1.7E5
40	Gallic acid	170	169	125	2.7/ 2.2E6	2.2/ 8.3E6	2.7/ 6.0 E5
41	Vanillic acid	167	166	152, 123	5.9/ 7.9 E5	5.5/ 2.1E5	6.5/ 1.9E5
42	Protocatechuic acid	154	153	109	4.7/ 1.5 E5	4.1/ 1.3E5	4.6/ 1.7E5

43	Syringic acid	197	196	182, 167, 153, 138	8.1/ 1.6 E5	7.9/ 1.3E5	8.0/ 1.4E5
44	p-coumarinic acid (4-Hydroxycinnamic acid)	164	163	119	8.5/ 1.9E5	-	8.5/ 2E5
45	Emodin	270	269	241, 225, 182, 197	29.1/ 2.0E5	28.5/ 1.1E5	28.9/ 1.1E5
46	Chrysophanol	254	253	253, 225, 210	28.2/ 1.0 E5	27.8/ 1.9E5	28.8/ 1.0E5
47	Phloridzin	436	435	273	19.8/ 2.4 E5	19.0/ 3.3E5	20.2/ 1.3E5
