# Supplementary material for: The Potential of the Inclusion of Prosopis farcta Extract in the Diet on the Growth Performance, Immunity, Digestive Enzyme Activity, and Oxidative Status of the Common Carp, Cyprinus carpio, in Response to Ammonia Stress
Source: Animals (Basel). 2025 Mar 20;15(6):895. doi: 10.3390/ani15060895 (PMC11939293; doi:10.3390/ani15060895)
Supplement: Supplementary file 1 [file animals-15-00895-s001.zip › Table S2.pdf]

Table S2: Compositions of vitamin and mineral premixes

Vitamin premix (per kg of diet)	vitamin A, 2000 IU; vitamin B1 (thiamin), 5 mg; vitamin B2 (riboflavin), 5 mg; vitamin B6, 5 mg; vitamin B12, 0.025 mg; vitamin D3, 1200 IU; vitamin E, 63 mg; vitamin K3, 2.5 mg; folic acid, 1.3 mg; biotin, 0.05 mg; pantothenic acid calcium, 20 mg; inositol, 60 mg; ascorbic acid (35%), 110 mg; niacinamide, 25 mg
Mineral premix (per kg of diet)	$MnSO_4$, 10 mg; $MgSO_4$, 10 mg; KCl, 95 mg; NaCl, 165 mg; $ZnSO_4$, 20 mg; KI, 1 mg; $CuSO_4$, 12.5 mg; $FeSO_4$, 105 mg; Co, 1.5 mg
